# Can Microplastics (MPs) Replace Conventional Mineral Aggregates? A Brief Review

**DOI:** 10.3390/polym18040505

**Published:** 2026-02-18

**Authors:** Min Ook Kim

**Affiliations:** Department of Civil Engineering, Seoul National University of Science and Technology, 232 Gongneung-ro, Nowon-gu, Seoul 01811, Republic of Korea; minookkim@seoultech.ac.kr

**Keywords:** microplastics, cementitious composites, aggregate substitution, interfacial bonding, mechanical performance, durability

## Abstract

Microplastics (MPs) are an increasingly pervasive pollutant, prompting interest in using them as a waste valorization feedstock in cementitious composites—most commonly as partial replacements for mineral aggregates. This review critically assesses the technical feasibility and implications of this approach based on current experimental and analytical evidence. Across the literature, MPs differ fundamentally from natural aggregates in stiffness, density, and surface chemistry, which weakens particle packing and interfacial bonding. Consequently, MP–aggregate substitution typically reduces workability and compressive strength and degrades durability-related performance, including resistance to chloride ingress, carbonation, and freeze–thaw action, with adverse effects generally increasing at higher replacement levels. While isolated benefits such as reduced unit weight and occasional post-cracking responses have been reported under specific mix designs, untreated MPs usually behave as mechanically inactive inclusions and stress concentrators rather than effective reinforcement. Major uncertainties remain regarding long-term durability and the risk of secondary MP release. Overall, MP-based aggregate replacement should be considered a conditional, application-specific strategy, currently most defensible for non-structural or function-driven applications under carefully defined performance and environmental criteria.

## 1. Introduction

The rapid proliferation of microplastics (MPs) has emerged as one of the most pressing environmental challenges of the twenty-first century, driven by the exponential growth of plastic production, insufficient recycling rates, and the progressive fragmentation of MPs in natural and engineered environments [1,2]. MPs—typically defined as plastic particles smaller than 5 mm—are now ubiquitously detected in marine, freshwater, terrestrial, and atmospheric systems, raising significant concerns regarding ecosystem health, human exposure, and long-term environmental persistence [2,3,4,5,6]. While substantial research efforts have focused on the detection, transport, and toxicological impacts of MPs, comparatively limited attention has been paid to their potential valorization as functional constituents in construction materials. And this imbalance has resulted in a critical gap between environmental risk mitigation strategies and material-level solutions capable of consuming MP waste at a scale commensurate with its generation.

Cementitious composites are considered one of the most material-intensive systems in modern society, consuming vast quantities of natural aggregates and contributing significantly to global resource depletion and carbon emissions [7,8]. In this regard, the partial or complete replacement of mineral aggregates with alternative materials has become an active area of research, particularly in relation to recycled aggregates, lightweight fillers, and polymer-based inclusions [9,10,11]. MPs, by virtue of their low density, polymeric nature, and wide variability in size, shape, and chemistry, present both an opportunity and a challenge when introduced into cement-based matrices. Unlike conventional aggregates, MPs are mechanically compliant, chemically inert in highly alkaline environments, and typically hydrophobic, all of which fundamentally alter load transfer mechanisms, interfacial bonding, and transport processes within hardened cementitious systems [12]. As a result, the question of whether MPs can truly replace aggregates cannot be answered through empirical strength measurements alone, but instead requires a mechanistic understanding of how MPs interact with cement hydration products across multiple length scales.

Existing studies on MP-modified cementitious composites report highly scattered and sometimes contradictory results with respect to mechanical strength, durability, and long-term performance. While reductions in compressive strength are frequently observed and often attributed to weak interfacial transition zones (ITZs) and increased porosity, other investigations report enhanced toughness, improved crack resistance, or superior freeze–thaw durability under specific mix designs and MP dosages [13,14,15,16,17]. These inconsistencies arise largely from the absence of unified material classifications, standardized testing methodologies, and mechanistic frameworks capable of linking MP properties—such as polymer type, particle morphology, surface characteristics, and volume fraction—to observed macroscopic performance. Consequently, many published works remain descriptive in nature, limiting their transferability to design practice and obscuring the conditions under which MPs may be beneficial, neutral, or detrimental to cementitious composites.

From a polymer science perspective, MPs introduce a fundamentally different reinforcement paradigm compared to conventional mineral aggregates [18,19,20]. Specifically, their viscoelastic behavior, low elastic modulus, and capacity for large deformation under stress suggest that their role is more analogous to that of energy-absorbing inclusions or crack-modulating agents rather than traditional load-bearing aggregates. Moreover, the hydrophobic surface characteristics of MPs have been shown to disrupt cement paste wetting and hydration continuity, resulting in modified interfacial chemistry and increased microstructural heterogeneity [20]. These competing mechanisms—namely stress redistribution versus interfacial weakening, and localized toughness enhancement versus amplification of transport pathways—necessitate a critical synthesis that transcends isolated experimental observations and instead emphasizes structure–property–performance relationships.

**Figure 1:**
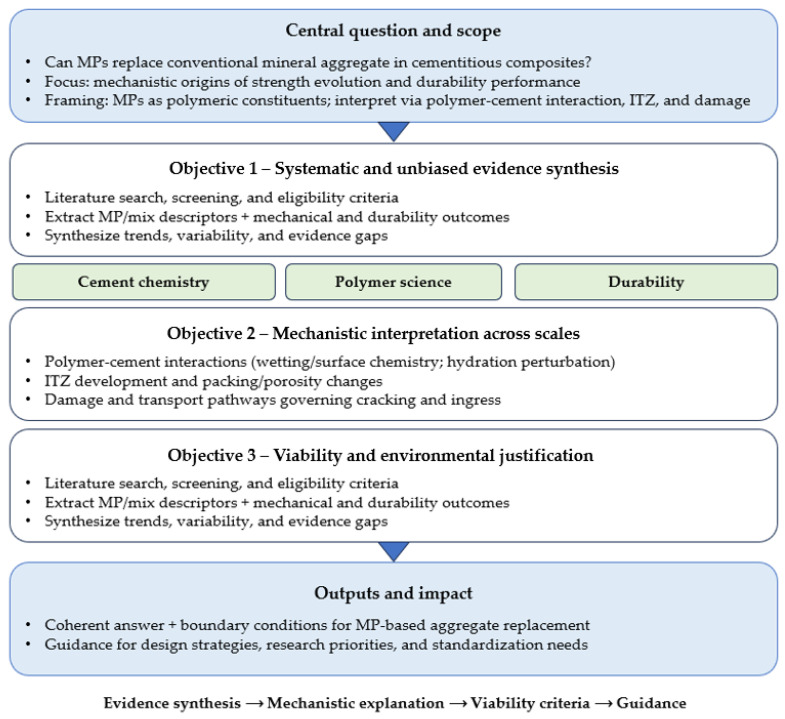
A schematic overview of the research workflow employed in this study.

Against this background, this study critically examines whether MPs can functionally replace conventional aggregates in cementitious composites, with a specific emphasis on the mechanistic origins of strength evolution and durability performance. Rather than framing MPs solely as recycled waste fillers, this review positions them as polymeric constituents whose behavior must be interpreted through the lens of polymer–cement interactions, interfacial transition zone development, and multi-scale damage mechanisms. The objectives of this review are threefold: first, to synthesize existing experimental evidence in a systematic and unbiased manner; second, to identify the dominant physical and chemical mechanisms governing mechanical and durability outcomes; and third, to delineate the conditions and application domains in which MP incorporation may be technically viable and environmentally justifiable. By integrating insights from cement chemistry, polymer science, and durability engineering, this review seeks to provide a coherent answer to the central question posed in the title: can MPs replace conventional aggregates, and if so, under what mechanistically defensible conditions? The findings aim not only to clarify current scientific understanding but also to inform future research directions, standardization efforts, and rational design strategies for next-generation cementitious composites incorporating polymeric waste streams. Figure 1 illustrates the research flow adopted in this study.

## 2. Microplastics as Polymeric Materials: Sources, Properties, and Classification

As mentioned previously, regardless of the exposure conditions they experience, MPs are inherently polymeric materials whose behavior in cementitious systems is governed by their intrinsic polymer chemistry, particle morphology, and surface physicochemical characteristics [2,21,22]. Unlike conventional mineral aggregates, therefore, MPs originate from synthetic polymers intentionally engineered to exhibit high durability, chemical stability, and resistance to environmental degradation, including ultraviolet radiation, moisture, and chemical attack [23,24]. As a result, MPs exhibit interfacial behaviors distinct from those of mineral aggregates, particularly with respect to hydrophobicity, surface energy, and bonding affinity with cement hydration products, which might significantly influence both the microstructure and performance of cementitious composites [1,10].

**Figure 2:**
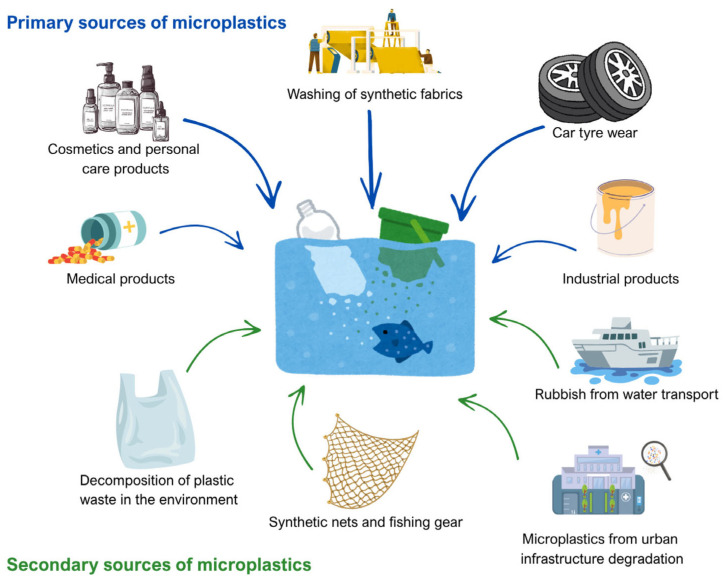
Sources of microplastics in aquatic environment, reprinted from Ref. [25].

MPs are commonly categorized according to their origin into primary and secondary microplastics [21,25,26]. Figure 2 illustrates the sources of microplastics in aquatic environments [25]. Primary MPs are intentionally manufactured at micro- to sub-millimeter scale for specific uses (e.g., polymer pellets/nurdles, cosmetic microbeads, and industrial abrasives). Their relatively controlled size and morphology can lead to more predictable packing and dispersion; in cementitious systems, these attributes mainly affect water demand/rheology, segregation resistance, and ITZ quality.

Secondary MPs, in contrast, form via fragmentation of larger plastics through abrasion, ultraviolet (UV)-driven photo-oxidation, thermal cycling, and chemical weathering, producing irregular fragments/fibers with heterogeneous sizes and often oxidized surfaces [25]. They dominate environmental and waste streams relevant to construction (e.g., packaging, textiles, and construction plastics) [26] and can therefore more strongly influence fresh and hardened behavior—especially workability, air void/pore structure, mechanical variability, and transport-related durability through altered ITZ and microcracking.

The origin of MPs is expected to strongly govern their physicochemical characteristics. This is because MPs derived from packaging plastics, synthetic textiles, automotive-related sources (e.g., tire/road wear), and construction/built-environment plastics (e.g., paints/coatings, sealants, polymer-modified materials) commonly exhibit heterogeneous polymer/additive compositions, irregular morphologies, and variable aging/oxidation states [27,28,29,30]. Accordingly, MPs considered for incorporation into cementitious composites should not be treated as uniform feedstocks; instead, they represent a diverse class of polymeric particulates whose performance is case-dependent and sensitive to particle source, geometry, surface oxidation, and contaminant/additive legacy [28,29,31].

From a polymer science perspective, the most frequently reported MPs consist of thermoplastic polymers such as polyethylene (PE), polypropylene (PP), polyethylene terephthalate (PET), polystyrene (PS), and polyvinyl chloride (PVC), as summarized in Table 1 [2,32,33].

These polymers can differ markedly in molecular structure, crystallinity, polarity, and glass transition temperature, which in turn govern their mechanical stiffness, thermal stability, and interfacial interactions with cementitious matrices. Specifically, non-polar polyolefins such as PE and PP exhibit low surface energy and pronounced hydrophobicity, resulting in weak physicochemical affinity toward hydrophilic cement hydration products [32]. In contrast, PET and PVC contain polar functional groups that can enhance interfacial adhesion through dipole–dipole interactions or limited hydrogen bonding [33]. Such differences imply that the performance of MPs in cementitious composites is not solely controlled by particle size or content, but also by polymer chemistry at the molecular level. Representative polymers in MPs have property-driven implications that must be considered before incorporation into cementitious composites

MPs are commonly defined as plastic particles <5 mm, yet in practice they span from sub-micron fragments to millimeter-scale particles, and this broad distribution governs packing, dispersion, and stress concentration in cementitious matrices [34,35]. When sufficiently fine and well dispersed, MPs may exhibit filler-like behavior that modifies rheology and pore structure, whereas larger particles behave more like lightweight aggregate analogs with limited load-bearing capacity and a greater propensity to create interfacial defects. Morphology adds a second controlling dimension, as MPs can occur as fragments, fibers, films, or near-spherical particles depending on source and degradation history [10,22,36]; fibrous forms may provide limited crack-bridging and post-peak toughness, while angular fragments more often act as stress raisers that trigger microcracking [36]. Because most polymers are less dense than mineral aggregates, segregation and mix heterogeneity become increasingly likely at higher replacement ratios, further amplifying variability in fresh and hardened performance [10]. These effects are typically accentuated for secondary MPs, which arise from fragmentation and weathering and therefore tend to be more polydisperse and surface-altered than primary MPs. Moderate aging can increase surface polarity and wettability, potentially reducing interfacial voids and encouraging local hydrate deposition, but it may also introduce leachable additives or degradation products that disturb hydration and generate weak boundary layers in the highly alkaline pore solution. Likewise, roughened and irregular surfaces can enhance mechanical interlocking when paste fully wets asperities, yet they more frequently increase water demand, promote agglomeration, and trap air within surface shadow zones, leading to a more porous ITZ and higher transport susceptibility. Overall, compatibility improves mainly when secondary MPs are predominantly fine-to-medium, moderately aged without substantial leaching, and uniformly dispersed at low dosages, whereas it deteriorates when coarse/irregular particles dominate or clustering/segregation and severe aging intensify workability loss and ITZ defects.

Unlike pristine polymers, environmentally derived MPs commonly exhibit altered surface chemistry as a result of photo-oxidation, hydrolysis, and mechanical abrasion during environmental aging processes, which fundamentally modify their physicochemical characteristics [23]. Such aging processes introduce oxygen-containing functional groups, increase surface roughness, and alter surface wettability, thereby significantly affecting interfacial interactions between MPs and cementitious hydration products. These changes may locally enhance mechanical interlocking or physicochemical bonding at the polymer–cement interface; however, the extent and nature of aging effects remain highly variable and difficult to control, resulting in substantial inconsistencies among experimental observations reported in the literature [37]. From a materials design perspective, this variability underscores the necessity of moving beyond treating MPs as inert fillers and instead adopting systematic surface classification or controlled surface modification strategies that exploit polymer aging effects in a reproducible and performance-oriented manner [38].

The polymeric nature of MPs distinguishes them fundamentally from conventional aggregates. Their chemical inertness toward cement hydration, low stiffness, and variable surface properties necessitate a paradigm shift in mix design philosophy. Rather than directly substituting mineral aggregates on a mass or volume basis, MPs should be regarded as polymeric inclusions, whose functions must be deliberately engineered through particle selection, surface treatment, and hybridization with other modifiers. This polymer-centric perspective provides a critical foundation for evaluating whether MPs can meaningfully replace aggregates, not as one-to-one substitutes, but as multifunctional components capable of tailoring mechanical response, durability, and sustainability when appropriately integrated into cementitious systems.

## 3. Incorporation of Microplastics into Cementitious Composites

**Figure 3:**
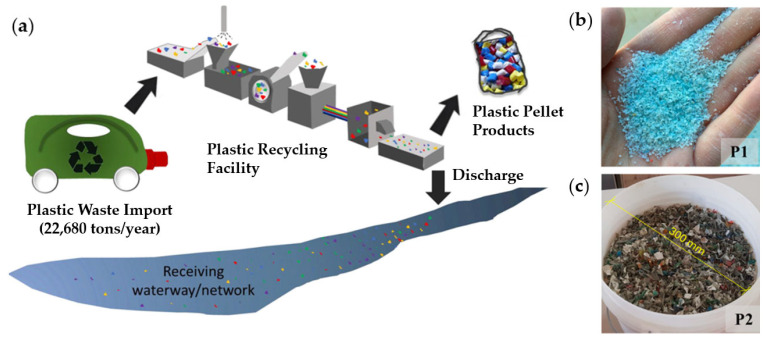
(**a**) Description of typical recycling process and plastic waste aggregates: (**b**) light-blue plastic flakes, P1; (**c**) plastic flakes, P2, adapted from Refs. [39,40].

Prior to incorporation into cementitious matrices, MPs are typically subjected to preprocessing steps to ensure basic compatibility with conventional mixing and casting operations, as shown in Figure 3 [39,40].

**Figure 4:**
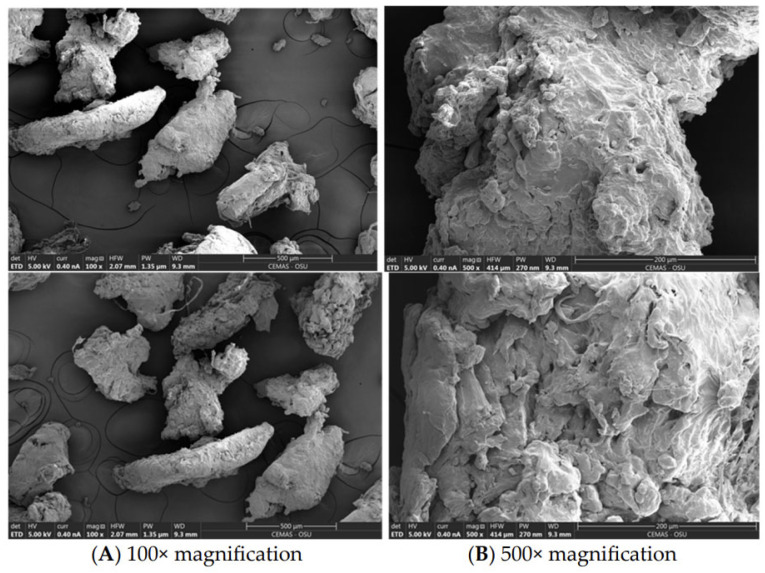
SEM images of grinded plastic waste particles (WPs), reprinted from Ref. [42].

These procedures commonly include washing to remove organic contaminants and soluble salts, drying to stabilize mass and volume, and sieving to control particle size distribution and reduce segregation during mixing [9]. However, these preprocessing steps can be resource-intensive and may contribute non-negligible environmental burdens, particularly through freshwater demand and wastewater generation during washing (requiring collection and treatment), and through thermal energy consumption and associated CO_2_ emissions during drying (e.g., oven- or hot-air drying). In several studies, MPs are produced directly from post-consumer plastic waste via mechanical grinding or shredding, while cryogenic milling has been employed to suppress thermal degradation and particle agglomeration during particle size reduction [41]. Notably, grinding/shredding requires electricity and can generate fine dust that necessitates filtration and safe handling, whereas cryogenic milling is typically more energy- and resource-demanding due to cooling requirements (e.g., liquid nitrogen production and use), which can substantially increase the life-cycle impacts of MP preparation despite improved particle quality. Figure 4 presents scanning electron microscopy (SEM) images of grinded plastic waste (PW) [42]. The particles exhibit an irregular morphology as shown in Figure 4A. The particles shown in Figure 4B have an approximate width of ~400 µm (0.4 mm) and present a rough surface with a spongy, porous structure.

Despite their widespread use, preprocessing protocols remain among the least standardized aspects of MP-modified cementitious composite research. Variations in cleaning methods, grinding energy, and size classification criteria yield MPs with inconsistent surface roughness and residual contamination levels, contributing to substantial scatter in reported mechanical and durability performance [43]. Moreover, untreated MPs typically retain intrinsically hydrophobic and low-surface-energy characteristics, which hinder cement paste wetting and promote interfacial voids and weak bonding zones. Although surface modification approaches—such as plasma treatment, chemical oxidation, and coating with mineral fines—have been proposed to enhance interfacial compatibility, their application remains largely limited to laboratory-scale investigations and has not yet been translated into standardized or scalable construction practices [44].

Achieving uniform dispersion of MPs within cementitious matrices remains a central technical challenge, as MPs tend to segregate, float, or agglomerate during mixing, particularly when introduced as dry aggregate replacements due to their low density and hydrophobic nature [45]. Such agglomeration leads to localized MP-rich zones, increased entrapped air, and discontinuities in the hardened matrix. Several mixing strategies have been explored to mitigate these effects [46,47]. Pre-dispersion of MPs in mixing water or admixture solutions can partially improve spatial distribution, although it may also increase water demand and reduce workability. Sequential mixing protocols, in which MPs are blended with cementitious fines prior to the addition of coarse aggregates, have been shown to enhance mechanical homogeneity in some cases [36]. The use of high-range water reducers (HRWRs) may further assist dispersion by reducing paste viscosity; however, excessive admixture dosages can adversely affect setting behavior and hydration kinetics. Notably, even when macroscopic dispersion appears satisfactory, microscopic examination often reveals weak interfacial zones surrounding MPs [48,49]. This observation underscores that dispersion alone is insufficient to ensure mechanical compatibility and that interfacial engineering remains a critical unmet need.

The incorporation of MPs exerts a pronounced influence on the fresh-state properties of cementitious composites, particularly workability, fresh density, and rheological behavior [50]. In the majority of reported studies, both slump and flow diameter decrease monotonically with increasing MP content [10,15]. This behavior is primarily attributed to the angular particle geometry, elevated specific surface area, and poor wettability of MPs, which collectively intensify internal friction and increase effective water demand. A concurrent and consistently observed outcome is the reduction in fresh density, reflecting the substantially lower specific gravity of polymeric MPs compared with conventional mineral aggregates [51]. Although such density reduction may be beneficial for lightweight construction applications, it simultaneously exacerbates the risk of segregation and bleeding, particularly in mixtures with insufficient paste volume or cohesion. Rheological investigations further demonstrate that MP incorporation generally increases both yield stress and plastic viscosity, with these effects becoming increasingly pronounced at higher replacement ratios [52]. Collectively, these fresh-state modifications impose practical constraints on allowable MP dosages and necessitate careful mixture redesign, especially for cast-in-place applications where adequate flowability and surface finishing quality are essential.

**Figure 5:**
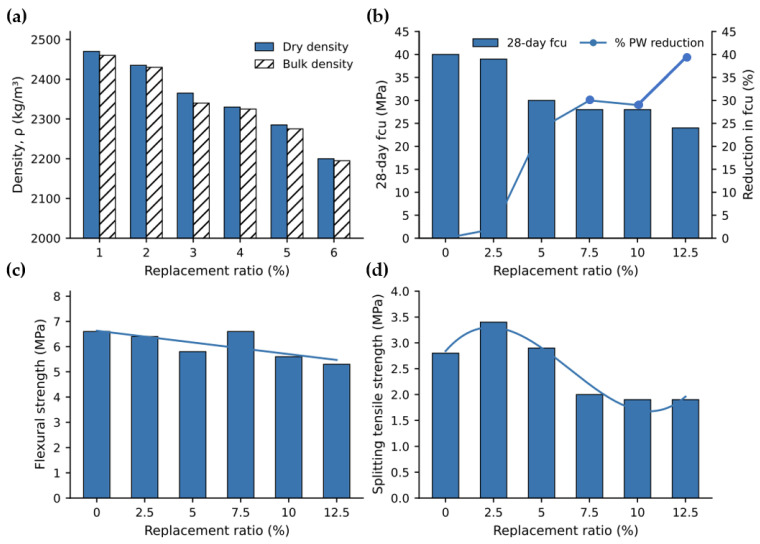
Effect of plastic waste replacement on measured concrete properties: (**a**) density; (**b**) 28-day compressive strength; (**c**) 28-day flexural strength; and (**d**) 28-day tensile strength, adapted from Ref. [55].

Mechanical performance constitutes the most extensively investigated aspect of MP-modified cementitious composites [53]. A broad consensus has emerged indicating that strength generally decreases with increasing MP content, largely independent of polymer type or particle geometry (see Figure 5) [20,54,55].

This degradation in strength is primarily attributed to three interacting mechanisms: (i) the introduction of mechanically compliant polymeric inclusions, (ii) increased porosity and entrapped air content, and (iii) weak interfacial bonding between MPs and the surrounding cementitious matrix [56]. In contrast, tensile and flexural responses exhibit more nuanced behavior.

When fibrous or elongated MPs are incorporated at low dosages, limited crack-bridging capacity and pull-out resistance may result in marginal enhancements in post-cracking behavior [10].

Nevertheless, such localized benefits are typically insufficient to offset the net reductions in stiffness and strength, particularly relative to conventional fiber-reinforced cementitious composites (FRCCs). Microscale and mesoscale investigations consistently indicate that MPs behave predominantly as stress concentrators rather than effective reinforcement elements [9]. Mechanistically, stress concentration arises from elastic and geometric incompatibility: low-modulus MPs embedded in a comparatively stiff cementitious matrix impose strain incompatibility under load, producing localized tensile/hoop stresses in the surrounding paste, especially at the poles of the inclusion aligned with the principal stress direction. When the MP–paste ITZ contains pre-existing defects (microvoids, incomplete hydration rims, or weakly adhered hydration products), the local interfacial traction demand exceeds the interfacial strength, triggering debonding at relatively small global strains; the debonded annulus then behaves mechanically like an internal flaw, further amplifying the stress intensity at the adjacent paste and accelerating crack initiation. Under mechanical loading, interfacial debonding initiates at the MP–cement paste interface, followed by crack propagation along the ITZ [57]. In addition, entrapped air and MP-induced porosity create void edge stress amplification; microcracks nucleated at multiple MP/void boundaries readily coalesce into a connected crack path, shifting failure from matrix-controlled to defect-controlled fracture. The absence of chemical bonding and the pronounced mismatch in elastic modulus between MPs and cementitious phases inhibit efficient stress transfer, ultimately promoting premature failure [36]. Collectively, these findings confirm that, in their untreated state, MPs function primarily as inert fillers with adverse mechanical consequences.

Overall, the effects of MPs on fresh and hardened properties indicate that their use in load-bearing structural concrete is presently unjustified. Across fresh-state performance, MPs frequently reduce workability and increase entrapped air/porosity, which can amplify batch-to-batch variability and undermine quality control. The systematic reduction in compressive strength and stiffness, together with the frequently reported increase in scatter, conflicts with the reliability requirements of structural design (e.g., characteristic strength targets, stiffness-controlled serviceability, and the assumptions implicit in partial safety factors). Moreover, the microstructural drivers identified in this section—elastic mismatch, weak MP–paste ITZ, early debonding, and void edge stress amplification—promote premature crack initiation and coalescence, explaining why losses in stiffness/strength are often disproportionate to the nominal replacement level. Instead, MP incorporation may be more appropriately considered for non-structural or semi-structural applications, where density reduction, waste valorization, or specific functional attributes are prioritized over mechanical performance. Examples include lightweight blocks and panels, insulating mortars/screeds, controlled low-strength materials (CLSMs) and backfills, vibration/noise-mitigating layers, and other products where performance criteria are governed by thermal/acoustic/impact response rather than high compressive capacity. Crucially, these conclusions should not be interpreted as a categorical rejection of MPs in cementitious composites, but rather as evidence that current implementation strategies remain fundamentally limited. Without deliberate interfacial engineering, controlled preprocessing, and application-specific mixture optimization, MPs cannot fulfill roles beyond that of low-performance aggregate substitutes. Accordingly, any credible pathway toward higher-value use requires (i) interfacial engineering (e.g., oxidation/plasma treatment, mineral/cementitious precoating) to improve wettability and adhesion; (ii) preprocessing/standardization (washing, grading, contamination control, shape control) to reduce heterogeneity; and (iii) mixture-level optimization (air management, packing/particle size distribution, supplementary construction materials (SCMs) synergy, and—where appropriate—hybrid reinforcement) to target functional gains without unacceptable reliability penalties.

## 4. Durability Performance, Environmental Implications, and Responsible Utilization Pathways

**Figure 6:**
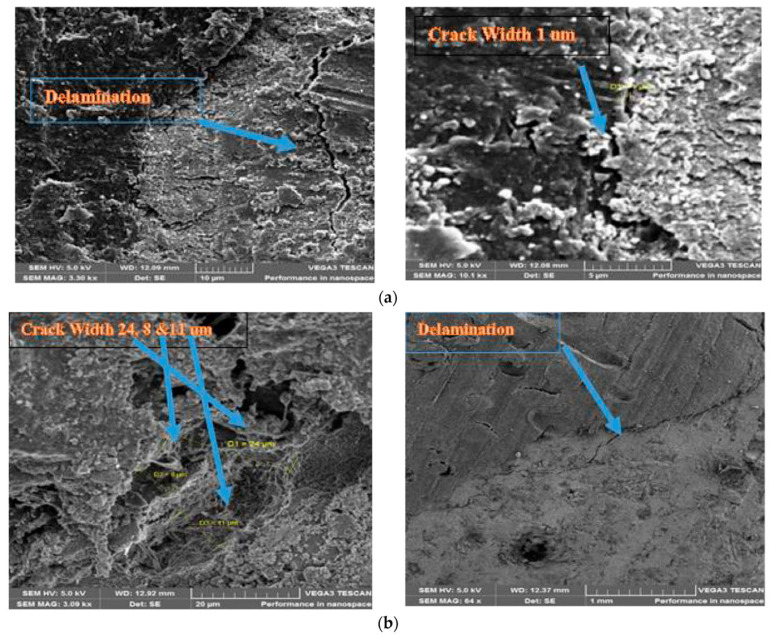
The crack width and bond pattern of natural and plastic aggregates with cement matrix: (**a**) natural aggregate concrete; (**b**) plastic aggregate concrete, reprinted from Ref. [59].

The incorporation of MPs into cementitious composites has a pronounced influence on transport-related properties, which directly govern durability performance [55]. Owing to their low stiffness, hydrophobic surface chemistry, and weak interfacial bonding with hydration products, MPs tend to disrupt the continuity of the cement matrix and increase total porosity, as illustrated in Figure 6 [58,59].

**Figure 7:**
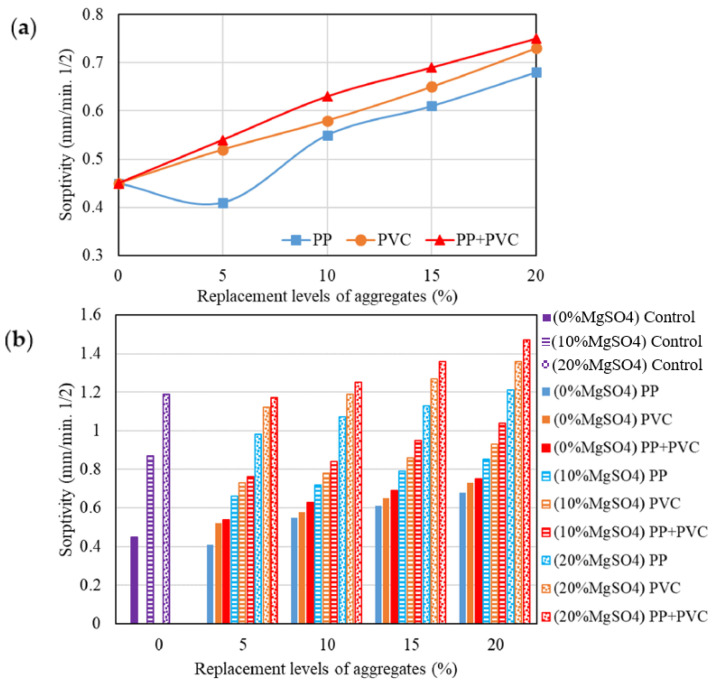
(**a**) Sorptivity results and (**b**) sorptivity results before and after exposure to MgSO4 attack, reprinted from Ref. [60].

Mercury intrusion porosimetry (MIP), water absorption, and sorptivity measurements reported in the literature consistently indicate an increase in connected pore volume with increasing MP content (See Figure 7) [60]. This altered pore structure adversely affects resistance to the ingress of aggressive agents, including water, chloride ions, and carbon dioxide [14]. As a result, elevated permeability and sorptivity can accelerate chloride penetration and carbonation depth, thereby increasing the risk of reinforcement corrosion in reinforced systems [42]. Similarly, the presence of MPs has been associated with reduced resistance to freeze–thaw cycles, primarily due to the increased availability of freezable water and the formation of preferential crack paths along MP–matrix interfaces [42].

While some studies have suggested that the deformability of certain MPs may locally alleviate stress concentrations under cyclic environmental loading, such effects remain secondary and highly dependent on MP morphology and dosage [61]. Overall, the prevailing evidence indicates that MP incorporation generally compromises durability-related performance indicators, particularly at moderate to high replacement levels.

Beyond conventional durability metrics, the long-term environmental stability of MPs embedded within cementitious matrices remains a critical yet still insufficiently resolved concern [27]. In principle, cement hydration products can physically encapsulate MP particles and thereby restrain their mobility—at least during the early stages of service life—by trapping them within a rigid, low-permeability solid matrix [62]. However, cementitious materials are inherently quasi-brittle and prone to cracking under combined mechanical loading and environmental actions (e.g., fatigue-related damage accumulation and cyclic exposure effects), which can create preferential pathways for transport and potential particle release [63,64]. In other words, crack formation, surface abrasion, or matrix degradation may progressively expose embedded MPs, raising the possibility of secondary microplastic release during service life or at end-of-life stages such as demolition and recycling. At present, systematic experimental evidence quantifying MP release from hardened cementitious composites is extremely limited. Moreover, no standardized testing protocols exist to evaluate MP liberation under realistic mechanical, chemical, or environmental stressors. This lack of data constitutes a significant knowledge gap. Without a rigorous understanding of long-term containment performance, the environmental justification for incorporating MPs into construction materials remains incomplete. Addressing this issue is essential for aligning material-level innovation with broader environmental protection objectives.

From a sustainability standpoint, the valorization of MPs in cementitious composites involves complex and often competing environmental considerations. On the positive side, diverting microplastic waste from landfills or natural ecosystems may reduce immediate environmental contamination and contribute to circular economy objectives [65,66]. Cementitious materials, owing to their large production volumes, offer a theoretically attractive sink for waste materials [67,68]. Conversely, the documented reductions in mechanical performance and durability may shorten service life or increase maintenance requirements, thereby offsetting potential environmental benefits through higher life-cycle emissions and resource consumption. Life-cycle assessment (LCA) studies suggest that environmental gains are highly sensitive to application context, MP dosage, and functional performance requirements [69,70,71]. Importantly, environmental benefits appear most plausible when MP incorporation is limited to non-structural applications where mechanical demands are modest and service conditions are controlled. In contrast, indiscriminate use in structural concrete risks undermining durability and sustainability objectives simultaneously. Therefore, MP utilization must be evaluated within a comprehensive life-cycle framework rather than through isolated material substitution metrics.

Recognizing the inherent limitations of untreated MPs, recent research has begun exploring functionalization strategies aimed at mitigating adverse performance impacts [13,14]. Surface modification techniques, including chemical oxidation, plasma treatment, and mineral coating, have been proposed to enhance interfacial adhesion between MPs and cement hydration products [72,73]. Hybrid approaches combining MPs with conventional fibers or reactive fillers have also been investigated to partially recover mechanical and durability performance [74]. While these strategies demonstrate conceptual promise, their scalability, cost-effectiveness, and environmental footprint still uncertain. Additional processing steps may introduce new environmental burdens that offset the benefits of waste utilization. Consequently, functionalization strategies must be evaluated not only in terms of material performance but also through holistic sustainability assessments.

While the incorporation of MPs into cementitious composites presents an intriguing opportunity for waste valorization, current evidence indicates substantial challenges related to durability, environmental risk, and long-term performance. MPs should not be viewed as conventional aggregate substitutes but rather as fundamentally distinct inclusions requiring careful engineering and application-specific justification. Responsible utilization will depend on a balanced assessment of material performance, environmental containment, and life-cycle impacts, supported by standardized testing and transparent sustainability metrics.

## 5. Synthesis, Limitations, and Future Research

The collective body of research reviewed in this study demonstrates that the incorporation of MPs into cementitious composites represents a conceptually attractive but technically constrained strategy for waste valorization [75,76]. At a fundamental level, MPs differ markedly from conventional mineral constituents in terms of stiffness, density, surface chemistry, and interfacial behavior. These intrinsic differences govern their influence on fresh-state behavior, mechanical performance, durability, and long-term environmental stability. Across a wide range of studies, a consistent pattern emerges: while MPs can be physically incorporated into cementitious matrices, their presence generally induces reductions in workability, compressive strength, and durability-related properties. These effects are not incidental but stem from fundamental micromechanical incompatibilities and weak interfacial bonding. Consequently, MP-modified cementitious composites cannot be evaluated using conventional aggregate replacement paradigms, nor can performance losses be dismissed as secondary or easily correctable. At the same time, the reviewed literature also indicates that the negative impacts of MPs are highly sensitive to polymer type, particle morphology, size distribution, dosage, and mixture design. This sensitivity suggests that MPs should be treated as design variables rather than waste fillers, requiring deliberate engineering rather than opportunistic substitution.

Despite rapid growth in the number of published studies, several methodological limitations continue to hinder meaningful comparison and generalization of results. First, the absence of standardized definitions and preprocessing protocols for MPs introduces substantial variability in reported properties. Differences in particle size ranges, surface contamination, and morphology often obscure mechanistic interpretation and contribute to conflicting conclusions. Second, most studies rely on short-term mechanical testing and basic durability indicators, with limited consideration of long-term performance in reinforced concrete under realistic service conditions. In particular, the implications of MP incorporation for reinforcement corrosion remain insufficiently resolved. It is expected that MPs may increase corrosion susceptibility indirectly by (i) elevating entrained air and connectivity of preferential pathways, (ii) increasing shrinkage and microcracking that accelerates chloride ingress and carbonation, and (iii) weakening the ITZ and paste–MP interfaces where damage localizes under sustained or cyclic loading. These mechanisms could shorten the time to corrosion initiation and amplify corrosion rate once depassivation occurs, especially in chloride-exposed environments. Accordingly, future work should move beyond compressive strength to corrosion-relevant endpoints, including chloride migration/diffusion, electrical resistivity, sorptivity/permeability, carbonation depth, corrosion potential mapping, and macro-cell/linear polarization resistance measurements, ideally under coupled cracking–chloride–wet–dry cycling and sustained load. Third, crack evolution, aging, coupled mechanical–environmental loading, and end-of-life scenarios remain largely unexplored. As a result, the long-term reliability and environmental safety of MP-modified reinforced concrete cannot yet be adequately assessed. Environmental evaluations are also frequently restricted to qualitative discussions of waste-diversion potential, with relatively few quantitative life-cycle or risk-based assessments. Without rigorous evaluation of service-life implications (including corrosion-driven maintenance demand) and potential MP release during cracking, abrasion, demolition, and recycling, claims of sustainability remain incomplete and, in some cases, premature. To mitigate potential long-term corrosion risks, research should prioritize interfacial engineering and mix design controls that restore transport resistance, e.g., limiting MP dosage and particle size extremes, incorporating SCMs to densify the pore network, optimizing air control and curing, and considering corrosion protection measures (corrosion inhibitors, increased cover depth, low-permeability overlays, or non-metallic reinforcement) for exposure-prone applications.

**Figure 8:**
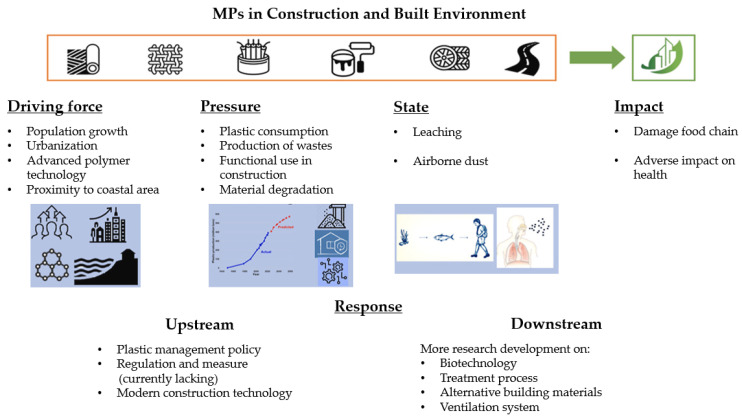
A DPSIR framework for microplastics in the construction and built environment, linking driving forces and pressures to environmental states, impacts, and upstream/downstream response strategies, adapted from Ref. [27].

Future research should move beyond generalized feasibility assessments and adopt an application-specific perspective [27,77,78,79,80,81,82]. Rather than asking whether microplastics (MPs) can be used in cementitious composites, the more pertinent question is under what conditions—and for which end uses—such incorporation can be technically robust and environmentally defensible. In the near term, the most credible pathways lie in non-structural applications, including lightweight panels, insulating layers, controlled low-strength materials, and sacrificial or replaceable components. In these contexts, moderate losses in mechanical performance may be tolerable when offset by clear functional benefits, such as reduced density, improved thermal insulation, or enhanced waste immobilization. By contrast, the use of MPs in structural concrete should remain highly restricted unless substantial advances are achieved in interfacial engineering, damage tolerance, and long-term durability control. To enable responsible deployment, it is essential to establish application-tailored performance thresholds that integrate mechanical performance, durability indicators, and environmental criteria, rather than relying on compressive strength as a single screening metric. Figure 8 summarizes these considerations using the DPSIR framework for MPs in the construction and built environment, linking driving forces and pressures to environmental states and impacts, and highlighting complementary upstream and downstream response strategies [27].

**Figure 9:**
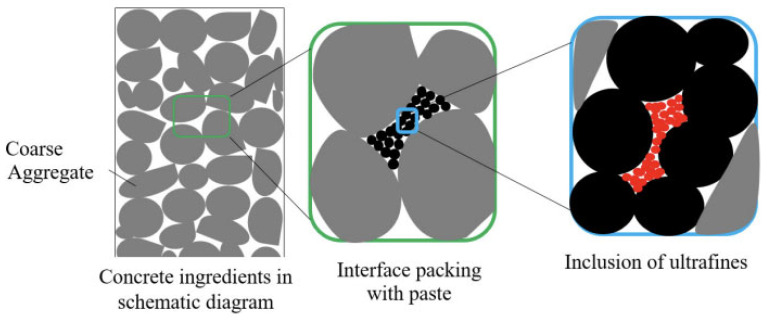
Schematic illustration of adding ultrafine fillers to improve packing of concrete, reprinted from Ref. [83].

Interfacial engineering is a critical leverage point for improving MP compatibility with cementitious matrices (Figure 9) [83]. Beyond single-parameter surface treatments, hybrid solutions—in which MPs are deliberately combined with complementary additives—offer a practical pathway to offset interfacial weakness while preserving workability and durability. Representative, application-relevant options include the following: (i) MP–SCM hybrid binders (e.g., MPs used alongside silica fume, metakaolin, or slag) to densify ITZ and reduce connectivity of capillary pores that exacerbate debonding; (ii) MP–fiber composite reinforcement (e.g., low-volume MPs co-dosed with microfibers such as PVA, basalt, or steel) to bridge shrinkage- and load-induced microcracks that preferentially initiate at MP-rich regions, thereby improving post-cracking response and mitigating strength scatter; and (iii) reactive or mineral-coated MP composites (e.g., thin coatings of silica/CaCO_3_, or cementitious/mineral fines anchored to the MP surface) to increase surface polarity/roughness and promote mechanical interlock and chemical affinity with hydration products. These hybrid strategies should be assessed holistically—not only for strength gains, but also for durability indicators (chloride transport, freeze–thaw scaling, water sorptivity) and for the net environmental balance. Critically, added processing must not undermine the sustainability rationale of MP utilization: performance improvements achieved via energy-intensive coating routes or high chemical demand may negate benefits from waste diversion. Accordingly, future work should prioritize low-impact, scalable hybrid designs (e.g., dry blending with widely available mineral fines or modest fiber additions) that are grounded in interfacial science rather than ad hoc optimization.

**Figure 10:**
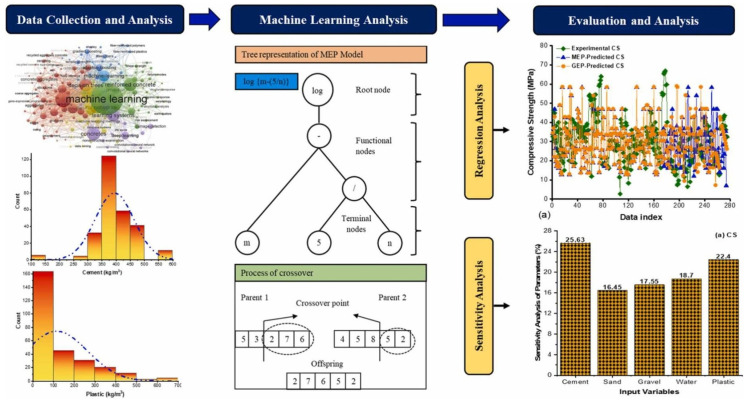
Data-driven programming for evaluating the mechanical properties of concrete containing plastic waste, reprinted from Ref. [84].

Artificial intelligence (AI) provides a practical, data-driven framework for managing the intrinsic variability and performance trade-offs of MP incorporation in cementitious composites (Figure 10) [84]. Recent studies on plastic-/MP-modified concretes demonstrate that AI can move beyond “trend fitting” to reliably predict and even optimize performance when polymer-related variables are explicitly encoded. For example, hybrid optimization–ML pipelines have combined particle swarm optimization (PSO) with Random Forest (RF) and XGBoost to predict compressive and tensile strengths of concretes containing plastic waste, showing that evolutionary search can be used to identify feasible mix-design regions under competing constraints (e.g., strength retention vs. plastic content) [85]. Likewise, literature-derived databases of recycled plastic aggregate concretes (including PET, PP, HDPE, PVC, etc.) have been modeled using SVR/RF/GBR/XGBoost families to predict 28-day compressive and flexural strengths, illustrating that tree-based ensembles can capture nonlinear interactions between polymer type, replacement ratio, and baseline mix parameters more effectively than simple regressions [86]. These examples directly motivate MP-specific workflows in which RF/boosting models are used not only for prediction, but also for feature importance ranking (e.g., identifying whether dosage, particle size/shape, or polymer type is the dominant driver of strength loss or transport resistance). In parallel, ANN/deep learning models are increasingly positioned to fuse heterogeneous inputs—mix proportions, curing histories, and microstructural descriptors (SEM/MIP/µCT-derived porosity, void connectivity, or crack metrics)—to predict macroscopic durability indicators with higher fidelity, while SVM-classifiers remain suitable for tasks such as MP-type discrimination or identifying threshold contents associated with unacceptable performance degradation.

The utilization of MPs in cementitious composites should be viewed neither as a universal solution to plastic pollution nor as an inherently flawed concept. Rather, it is a conditional strategy whose viability depends on rigorous engineering, transparent environmental assessment, and application-specific justification. To accelerate translation beyond exploratory demonstrations, future research should explicitly aim to engineer MPs to behave more like traditional mineral aggregates in both fresh and hardened states. Concretely, this includes (i) particle morphology control (e.g., producing angular, graded particles and limiting excessive flatness) to improve packing and mechanical interlock; (ii) surface “mineralization” or roughening via low-impact coatings (silica/CaCO_3_/cementitious fines) to increase surface polarity, wettability, and ITZ cohesion; (iii) density and stiffness tuning through composite or filled MPs (e.g., incorporating mineral fillers) to reduce modulus mismatch and stress concentrations; and (iv) pre-saturation or moisture management protocols and admixture compatibility screening to minimize entrapped air and workability loss. These directions should be embedded within standardized, performance-driven frameworks that benchmark engineered MPs against reference aggregate gradations and durability targets (transport, freeze–thaw, abrasion), alongside cradle-to-gate LCA to verify that added processing does not negate sustainability gains. Only through such an engineering-led and sustainability-informed transition can MP utilization in cementitious materials evolve from an experimental curiosity into a responsible and credible component of sustainable construction practice.

To make sustainability claims verifiable, future studies should report application-specific LCA case studies for MP-modified concretes, explicitly quantifying trade-offs between (i) waste diversion and reduced virgin aggregate demand and (ii) performance-driven penalties (e.g., higher cement content, additional processing, shorter service life). A practical way forward is to adopt a consistent functional unit (e.g., 1 m^3^ of concrete meeting a target strength/durability class or 1 m^2^ of pavement delivering a defined design life), define system boundaries (A1–A4 at minimum, and ideally use-phase and maintenance when durability changes), and include sensitivity analyses for transport distance, MP preprocessing energy, and mix redesign (cement/SCM and admixtures).

Concrete-relevant LCA examples already illustrate these trade-offs. For instance, an LCA of plastic-waste-based paver blocks compared alternative plastic recycling/manufacturing routes and showed that environmental benefits are highly contingent on the processing pathway and energy demand—highlighting that waste utilization does not automatically translate into lower impacts [87]. Similarly, a recent LCA on incorporating HDPE plastic milk-bottle waste into residential concrete evaluated both mechanical performance and environmental impacts, demonstrating that benefits depend on replacement level and mix design (e.g., whether additional binder or modifiers are required to recover strength) [88]. Complementary evidence from a comparative LCA conference study that examined concretes incorporating recycled plastic (PET/HDPE), rubber, and recycled aggregates further emphasizes that outcomes can flip with assumptions about functional unit and durability equivalence—reinforcing the need for standardized reporting and scenario-based analysis [89].

Building on these precedents, MP-modified concrete studies should move beyond cradle-to-gate CO_2_ only and explicitly evaluate (1) mix redesign burdens (extra cement/chemicals needed to meet performance targets), (2) durability-adjusted service life (chloride ingress/carbonation and maintenance frequency), and (3) end-of-life fate (crushing/recycling and potential MP release).

## 6. Conclusions

This review shows that incorporating MPs into cementitious composites is constrained mainly by fundamental material incompatibilities, not merely by mix design limitations. Because MPs exhibit low stiffness and density, hydrophobic surface chemistry, and weak bonding with cement hydration products, they cannot be treated as conventional aggregate substitutes. Across the literature, their inclusion most often reduces workability, mechanical performance, and durability, with adverse effects becoming more pronounced as replacement levels increase.

While some studies report isolated advantages—such as lower density or marginally improved post-cracking deformation—these benefits are highly conditional and strongly dependent on MP type, size, shape, surface condition, and dosage. In their untreated state, MPs largely act as mechanically inactive inclusions and stress concentrators, which provides no robust foundation for use in load-bearing structural concrete.

From an environmental perspective, the proposition that cementitious materials can serve as effective sinks for microplastic waste remains unresolved. Any potential benefit from immobilizing plastic waste may be counterbalanced by reduced service life, higher maintenance demand, and an uncertain risk of secondary MP release during service, demolition, recycling, or disposal. The lack of standardized methods to evaluate long-term containment and release further limits confident environmental appraisal.

Accordingly, MP utilization should be framed as a conditional, application-specific strategy that is currently defensible primarily for non-structural or function-driven uses, and only under clearly defined performance and environmental criteria. Within these limits—and only with rigorous validation—MP incorporation could contribute in a limited, controlled manner to sustainable construction practices.

Future studies are necessary to (i) establish standardized mix design, testing, and reporting protocols that enable meaningful cross-study comparison; (ii) quantify ITZ mechanisms through multi-scale characterization and micromechanical modeling, including the role of MP morphology, aging, and surface chemistry; (iii) develop and assess surface treatments or compatibilizers that improve bonding without introducing unacceptable cost, toxicity, or durability penalties; (iv) determine dose–response thresholds and performance envelopes for specific application classes (e.g., lightweight non-structural panels, insulating renders, acoustic layers); (v) evaluate long-term durability under realistic coupled exposures (wet–dry, freeze–thaw, chloride ingress, carbonation, sulfate attack, UV/thermal cycling) using accelerated-to-field correlation; (vi) investigate secondary MP release pathways during service, abrasion, cracking, sawing, and end-of-life processing, supported by validated capture and quantification methods; and (vii) conduct full life-cycle and risk assessments that integrate service life modeling, maintenance scenarios, and end-of-life fate to determine whether MP incorporation yields a net environmental benefit relative to alternative waste management routes.

## Figures and Tables

**Table 1:** Representative Thermoplastic Polymers Commonly Identified in Microplastics and Their Key Physicochemical Properties Relevant to Cementitious Composites [2,32,33].

Type	Typical Density (g·cm^−3^)	Glass Transition/ Melting Temp. (°C)	Surface Polarity/ Hydrophobicity	Elastic Modulus (GPa)	Key Characteristics	Implications for MP Incorporation into Concrete (Property ⟶ Effect)
PE	0.91–0.96	Tg ≈ −125 Tm ≈ 110–135	Highly non-polar, strongly hydrophobic	0.2–1.5	Low surface energy and chemical inertness result in weak interfacial bonding with cement hydration products	Hydrophobic + low surface energy ⟶ poor wetting by pore solution and weak ITZ adhesion Low modulus ⟶ acts as soft inclusion lowering stiffness/strength Low density ⟶ increased floating/segregation risk
PP	0.90–0.91	Tg ≈ −10 Tm ≈ 160–170	Non-polar, hydrophobic	1.3–2.0	Higher stiffness than PE but similarly poor wettability; contributes to reduced mechanical performance and increased air entrainment	Hydrophobic surface ⟶ limited chemical interaction with hydrates Low density ⟶ segregation/floating tendency during casting Moderate modulus ⟶ reduce compressive strength but may improve impact resistance depending on shape (e.g., fibers) and dosage
PS	1.04–1.06	Tg ≈ 95–105	Weakly polar, hydrophobic	2.5–3.5	Rigid but brittle polymer; limited mechanical compatibility and weak interfacial adhesion with cement paste	High Tg + brittleness ⟶ particles behave as rigid/brittle inclusions; can initiate stress concentrations and microcracks under load Hydrophobicity ⟶ weak ITZ
PET	1.34–1.39	Tg ≈ 70–80 Tm ≈ 250–260	Moderately polar	2.5–4.0	Presence of ester functional groups enhances surface polarity and potential physicochemical interaction with cement hydrates	Moderate polarity (ester groups) ⟶ improved wetting/dispersion and potentially stronger ITZ than polyolefins Higher density ⟶ reduced segregation and more stable particle distribution Higher modulus ⟶ less soft-inclusion effect, but irregular shapes can still degrade strength if ITZ/voids are not controlled
PVC	1.30–1.45	Tg ≈ 80–90	Polar due to C–Cl bonds	2.5–4.0	Higher density and polarity improve dispersion stability; potential for limited interfacial adhesion compared to polyolefins	Higher polarity ⟶ better wettability and dispersion than PE/PP, with potential for improved ITZ continuity Higher density ⟶ low flotation risk
PA	1.12–1.15	Tg ≈ 40–70 Tm ≈ 215–265	Polar, hydrophilic tendency	2.0–3.0	Amide functional groups promote hydrogen bonding; comparatively improved compatibility with hydrophilic cementitious matrices	Hydrophilicity + amide groups ⟶ strong wetting and higher likelihood of hydrogen bonding with hydration products, improving ITZ relative to hydrophobic MPs; may increase water demand (surface adsorption) and affect rheology

## Data Availability

No new data were created or analyzed in this study. Data sharing is not applicable to this article.

## Abbreviations

The following abbreviations are used in this manuscript:

MPs	Microplastics
ITZs	Interfacial Transition Zones
UV	Ultraviolet
PE	Polyethylene
PP	Polypropylene
PET	Polyethylene Terephthalate
PS	Polystyrene
PVC	Polyvinyl Chloride
PW	Plastic Waste
SEM	Scanning Electron Microscopy
HRWRs	High-Range Water Reducers
FRCCs	Fiber-Reinforced Cementitious Composites
CLSM	Controlled Low-Strength Material
SCM	Supplementary Cementitious Material
MIP	Mercury Intrusion Porosimetry
LCA	Life-Cycle Assessment
AI	Artificial Intelligence
RF	Random Forest
ANN	Artificial Neutral Network
SVMs	Support Vector Machines
